# 

**Published:** 1813-02

**Authors:** 


### MONTHLY CATALOGUE OF MEDICAL BOOKS.

OBSERVATIONS on the Nature and Cure of Dropsies, and particularly on the Presence of the coagulable part of the Blood in Dropsical urine; to which is added an Appendix, containing several Cases of Angina Pectoris, with Dissections, &c. By John Blackhall, M. D. 8vo.—Longman and Co.

Medico-Chirurgical Transactions, published by the Medical and Chirurgical Society of London. Volume the Third. 8vo.—Longman and Co.

Companion to the London Dissector, or the Art and Method of making Preparations, exhibiting the Structure of the Human Body. 12mo.—Cox.

An Introduction to Medical Literature, including a System of Practical Nosology, intended as a Guide to Students and an Assistant to Practitioners. By Thomas Young, M.D. F.R. and L.S. 8vo.

---

### *NOTICES TO CORRESPONDENTS.*

*Besides some American Papers from Drs. Hosack and Francis, Communications have reached us from Dr. A. B. Granville; Messrs. G. N. Hill, J. A. Bradley, R. Semple, &c. &c.*

*Mr. Stevenson's reply to Dr. Farre came too late for insertion this month.*

*We request that authors who wish for an early notice of their Works, will direct their publishers to forward them to Mr. Adlard's Printing Office, No. 23, Bartholomew Close, or to No. 1, Paternoster Row, addressed to the Editors of the Medical and Physical Journal.*

*The Report from the Small-pox Hospital we are obliged, for want of room, to defer until the publication of our next Number.*

